# Narrative Review of Low-Intake Dehydration in Older Adults

**DOI:** 10.3390/nu13093142

**Published:** 2021-09-09

**Authors:** Anne Marie Beck, Johanna Seemer, Anne Wilkens Knudsen, Tina Munk

**Affiliations:** 1 Department of Dietetic and Nutritional Research, Copenhagen University Hospital Herlev and Gentofte, DK-2730 Herlev, Denmark; 2 Institute of Nursing and Nutrition, Faculty of Health, University College Copenhagen, DK-1799 Copenhagen, Denmark; anne.wilkens.knudsen.01@regionh.dk (A.W.K.); tina.munk@regionh.dk (T.M.); 3 Institute for Biomedicine of Aging, Friedrich-Alexander-Universität Erlangen-Nürnberg, 90408 Nuremberg, Germany; johanna.seemer@fau.de

**Keywords:** osmolarity, osmolality, interventions, drinks, hydration

## Abstract

Low-intake dehydration is a common and often chronic condition in older adults. Adverse health outcomes associated with low-intake dehydration in older adults include poorer cognitive performance, reduced quality of life, worsened course of illness and recovery, and a high number of unplanned hospital admissions and increased mortality. The subjective methods to assess (risk of) dehydration are not reliable, and the evidence about preventive measures are also limited. So is the knowledge about the optimal intake of beverages per day. This narrative review presents the state of the science on the role of low intake hydration in older adults. Despite its simple cause—the inadequate intake of beverages—low-intake dehydration appears to be a very complex problem to address and much more research is needed in the area. Based on the existing evidence, it seems necessary to take setting specific differences and individual problems and needs into account to tackle dehydration in older adults. Further, it is necessary to increase awareness of the prevalence and severity of low-intake dehydration among older adults and in nursing staff in care homes and hospitals as well as among caregivers of older adults living at home.

## 1. Introduction

Low-intake dehydration is often referred to as hypertonic, hyperosmotic, or water-loss, and describes an uncompensated, predominantly pure water deficit [1]. Low-intake dehydration is commonly caused by a beverage intake insufficient to compensate potential fluid losses through urine, feces, breath, and/or sweat [2]. This leads to a concentration of particles within body fluids, a decrease in extracellular fluid volume, and an increase in directly measured serum osmolality [3].

Low-intake dehydration is a common often chronic health condition in older adults [1,2,3]. When using the measurement of serum osmolality, several studies have found that the prevalence of low-intake dehydration is lowest among older adults living at home, increased in long-term care residents, and highest in older hospitalized adults [1,3,4]. The high prevalence of dehydration in long-term care residents has been confirmed in a recent systematic review [2]. Specifically, dysphagia, which is prevalent in about one in seven nursing home residents [5], is a major risk factor for poor outcomes including low-intake dehydration [6]. Regarding the hospital setting, studies with admission data of older patients reflect their increased risk of low-intake dehydration and show increased osmolality in almost 50% [1]. Unfortunately, data on low-intake dehydration developing during hospitalization are sparse [4]. However, it has been reported that two-thirds of patients that were dehydrated on admission, were still dehydrated after 48 h [1].

There are several reasons for a high prevalence of low-intake dehydration among older adults: With age, there is a weakening of physiological mechanisms after insufficient fluid intake that may increase the risk of low-intake dehydration, including a decrease in thirst sensation and primary urine concentration by the kidney [4]. In addition, total body water decreases with age, resulting in lower fluid stores. This aspect is exacerbated by the frequent use of diuretics and laxatives [4]. Besides physiological causes, low-intake dehydration in older people can be caused by a range of other risk factors, such as polypharmacy [7], functional and cognitive impairment [3,8], and voluntarily reduced fluid intake: This voluntary reduction can be for a variety of reasons, ranging from the assumption that intake is sufficient for their health to fear of incontinence; social isolation; reduced physical function and access to beverages; as well as swallowing problems and dysphagia [4].

Adverse health outcomes associated with low-intake dehydration in older adults are multifaceted, ranging from poorer cognitive performance, reduced quality of life, delirium, falls, fractures, worsened course of illness and recovery to heart disease, heat stress, kidney failure, unplanned hospital admissions, and increased mortality [8,9]. As low-intake dehydration is associated with poorer health and thus increased medical treatments, it is not surprising that experts agree that low-intake dehydration in older adults entails high costs for the health systems [9]. Low-intake dehydration and the associated consequences have repeatedly been shown to be a major cause of avoidable hospital admissions in the US and Europe, and it has been estimated that this leads to an economic burden in the US of USD 5.5 billion (in 2004) [1].

A major problem regarding prevention and tackling of low-intake dehydration in older adults is that assessment methods widespread in clinical practice (e.g., skin turgor, urine color and volume, heart rate, feeling of a dry mouth, thirst sensation) are not reliable [8]. Some clinical signs associated with low-intake dehydration may be misleading as they can be consequences of other conditions common in this age group. Symptoms like tongue furrows, dry mucous membranes, and urine specific gravity, may indicate low-intake dehydration, but may also be influenced by medications [10]. Another problem is that the evidence on preventive measures is limited, as is knowledge about the optimal intake of beverages per day for older adults [4].

These aspects were examined in systematic literature searches for the European Society for Clinical Nutrition and Metabolism (ESPEN) guideline on nutrition and hydration in geriatrics [4]. This resulted in several evidence-based recommendations focusing on low-intake dehydration. The aim of this narrative review is to present the evidence-based recommendations on low-intake dehydration by ESPEN and based on a literature review, check if new evidence is consistent/inconsistent with the ESPEN recommendations and identify remaining gaps to research on the role of low-intake hydration in older adults.

## 2. Materials and Methods

The evidence-based recommendations in the ESPEN guideline are based on several systematic literature searches, comprising a total of 33 PICOs (Participant, Intervention, Comparator, Outcomes, study design), which were finally split into four chapters. One of these dealt with the topic ‘‘Recommendations to prevent, identify and treat dehydration” [4]. The identified literature was graded according to the level of evidence, and accordingly, the grades of the recommendations were decided (i.e., level A indicated by ‘‘shall’’, level B by ‘‘should’’ and level 0 by ‘‘can”. The Good Practice Point was based on expert opinions due to lack of studies). The results were 82 recommendations, including both nutrition and hydration, where approximately half were graded with A, B, or 0 [4].

For the present narrative review an update of the literature was performed by the first author (AMB) in a three-step approach: (Step 1) Searches for guidelines that address low-intake dehydration and which is not included in the evidence-based recommendations have been updated until 10 April 2021 using Google Scholar and PubMed; (Step 2) Searches for more recent systematic reviews and primary studies were updated to 10 April 2021 by opening each of the references included in the evidence-based recommendations focusing on low-intake dehydration and undertaking a search of ‘‘similar articles’’ in PubMed. Searches in PubMed were limited to participants 65 years or older and publication date not older than July 2016, when the systematic literature searches for the ESPEN guideline ended, (Step 3) In addition, abstracts from the most recent ESPEN conference (2020) taking place after the release of the ESPEN guideline, were screened to look for topics in relation to low-intake dehydration. Only recommendations with a level of evidence A or B focusing on specific low-intake dehydration (see an overview in Table 1) were included in the search for more recent literature. ESPEN guideline contain two more evidence-based recommendations regarding treatment of low-intake dehydration (recommendation 72 and 73). The recommendation is to offer subcutaneous or intravenous fluids. As this is often not feasible outside the hospital, these two recommendations were not included.

**Figure 1:**
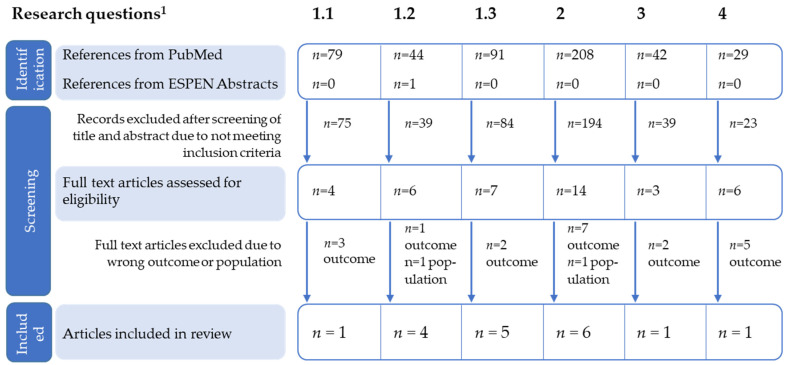
Flow chart of the identification of new studies according to each research question. ^1^ Six different searches were conducted in total (three searches for research question (RQ). RQ1: How should low-intake dehydration be identified in older persons? 1.1 by osmolality, 1.2 by osmolarity, 1.3 by clinical signs and bioelectrical impedance. RQ2: What interventions may help to support older persons to drink well and prevent low-intake dehydration? RQ3: How much should older people drink each day? RQ4: What should older people drink each day?

First, the literature search and an initial screening of titles were performed, followed by a screening of abstracts and full text. This was conducted for each recommendation listed in Table 1 and with the use of the same research questions that were defined and operationalized in the ESPEN guideline. These research questions are also presented in Table 1. For research question 1, three searches were conducted to adequately identify relevant studies, resulting in six searches in total (see Figure 1). Studies in a non-English language, without available abstract or full-text and not involving the relevant population, intervention, or outcome were excluded. When a full text was included, the consistency/inconsistency with the ESPEN recommendations within the specific area was evaluated, e.g., did the result of the paper supported the recommendation against the use of simple signs and tests, or were there any redundancies. The type of study was noted, e.g., whether it was a review an expert opinion, an observational study, a diagnostic accuracy study or a pre-post study. In addition, the quality of the studies was rated with the grading system of the Scottish Intercollegiate Guidelines Network (SIGN), which checklists was also used in the original ESPEN guideline

## 3. Results

### 3.1. Literature Search

A flowchart documenting the updated search strategy and results for each research question (Table 1) is shown in Figure 1 (three separate searches were conducted for research question 1, resulting in six different searches). To describe the current state of recommendations, the newly identified studies are shortly displayed in Table 2 and included in the different research questions below.

### 3.2. How Should Low-Intake Dehydration Be Identified in Older Persons?

As can be seen in Table 1, the ESPEN guideline recommends assessing low-intake dehydration by using objective measures. To objectively assess hydration status, plasma osmolality (the concentration of solutes in the blood) is a valuable parameter and might be considered a gold standard to detect low-intake dehydration in clinical practice [25]. For example, the US Institute of Medicine stated several years ago that plasma osmolality was the primary marker of low-intake dehydration levels [26].

- Osmolality

Regarding the use of serum-osmolality to identify low-intake hydration, the literature search identified one additional study:

In a multidisciplinary consensus on dehydration published in 2019 and based on a modified Delphi approach, one of the key messages was that plasma osmolality represents an important, objective marker of low-intake dehydration, which is rarely used in clinical practice [1]. Hence this supports the recommendations from ESPEN.

- Osmolarity

Regarding the use of the osmolarity equation, the literature search identified four additional studies:

The already mentioned multidisciplinary consensus on low-intake dehydration published in 2019 by Lacey et al. [1] supported the ESPEN guideline by the following recommendation: If direct measurement of serum/plasma osmolality is not possible, the authors recommend the calculation of plasma osmolality as a surrogate (see Table 1).

In the observational study of Munk and co-authors [11], 90 older patients from the emergency medical department were included. The aim was to validate the agreement between measured serum-osmolality (mOsm/kg) and calculated serum-osmolarity (mOsm/L). According to osmolality, impending dehydration was identified in 29 (32%) participants. There was a significant correlation between osmolality and osmolarity (r^2^ = 0.7513, *p* <0.001). The authors observed a sensitivity of 90%, a specificity of 68%, a positive predictive value of 26%, and a negative predictive value of 98%. In this study, only 20% of the patients who were dehydrated according to osmolality were correctly diagnosed with low-intake dehydration by clinical signs [11].

A recent study by Wojszel [12] also chose to use calculated osmolarity (see Table 1) and assessed the correlation between this measure and different patient characteristics. In 209 (58%) patients impending dehydration (>295 mmol/L) was detected at admission to the geriatric department. Impeding dehydration was more frequent in patients with multimorbidity, different chronic diseases and polypharmacy [12], which are well-known risk factors for low-intake dehydration assessed by measured serum osmolality and described in the ESPEN guideline [4].

Low-intake dehydration was also assessed by means of the osmolarity equation in the Berlin Aging Study. Based on longitudinal data from this study, the authors concluded that low-intake dehydration predicts a longitudinal decline in cognitive functioning and well-being among community-dwelling older (≥60 years) adults, [13]. This are well-known consequences of dehydration [4,8]. Among the 1047 participants with available data on all variables of interest during the largest assessment wave, 345 (33%) were identified with low-intake dehydration (osmolarity > 296 mmol/L) [13].

- Clinical signs and bioelectrical Impedance (BIA)

As can be seen in Table 1, the ESPEN guideline does not recommend clinical signs or use of Bioelectrical Impedance (BIA) to assess low-intake dehydration.

These recommendations are mainly based on a Cochrane review comparing non-invasive methods of fluid assessment status in older people which concluded that neither was reliable when compared to serum osmolality [8].

Regarding the recommendations against using subjective measures or BIA to assess low-intake dehydration, the literature search identified five new studies, which all examined clinical signs:

In the study from Bunn and Hooper [14], 49 signs and symptoms of low-intake dehydration were assessed. Low-intake dehydration was assessed by means of serum osmolality. Signs and symptoms included skin turgor; mouth, skin, and axillary dryness; capillary refill; sunken eyes; blood pressure on resting and after standing; body temperature; pulse rate; and self-reported feelings of thirst and well-being. None of the 49 commonly used clinical signs and symptoms was adequate to distinguish between hydrated participants and individuals with low-intake dehydration. They suggested that these assessments should be withdrawn from clinical practice and replaced by a 2-stage screening process. First, serum osmolarity (see Table 1) should be determined. Second, if calculated serum osmolarity is >295 mmol/L, there is a risk of dehydration, and measurement of serum osmolality is indicated [14].

The above mentioned multidisciplinary consensus on dehydration [1], supported both the work from Bunn and Hooper and ESPEN by the following recommendation: Outside of extremes, clinical signs, and symptoms of low-intake dehydration in adults may be unreliable. Clinical signs and symptoms should not be used alone to assess hydration status [1].

The recommendation was additionally confirmed by the study of Johnson and Hahn [15] performed among 60 nursing home residents. The authors found that clinical signs of mucous membranes and tongue furrows did not correlate with serum osmolality [15].

That the use of fluid intake as a clinical sign of low-intake dehydration is not related to serum osmolality was confirmed in another study: Akdeniz and co-authors [16] conducted an observational study in a geriatric hospital. They measured, among others, 3-day fluid intake and serum osmolality. Forty patients were included with a mean fluid intake of 1747 mL/day. Despite the relatively high intake of fluids, 20% of the patients were dehydrated and 23% had an impending dehydration according to serum osmolality. As a result, the authors conclude by questioning the clinical relevance of the fluid intake measure [16] and hence lend support to the recommendation from ESPEN.

In contrast to these findings are the results of another study in 38 older patients admitted to a geriatric department for rehabilitation after hip fracture surgery [17]. High serum osmolality (≥300 mOsm/kg) that correlated with clinical signs, was present in seven patients, i.e., presence of tongue furrows and poor skin turgor. Additionally, patients with hyperosmolality drank less than the others (median 785 vs. 1044 mL), but this difference was not significant [17].

### 3.3. What Interventions May Help to Support Older Persons to Drink Well and Prevent Low-Intake Dehydration?

To prevent low-intake dehydration, the ESPEN guideline recommends implementing multicomponent strategies for all residents living in residential care. These strategies should include high availability, choice, and frequent offering of fluid. In addition, staff should be sensitized to the need for adequate hydration and to support older adults to drink and to go to the toilet quickly when needed [4].

Further, the guideline recommends that strategies to support adequate hydration should be developed with the involvement of all relevant stakeholders, including older persons, staff, management, and policymakers [4].

In general, Volkert and co-authors concluded that no intervention was clearly able to prevent or treat low-intake dehydration and that there was limited information on increasing fluid intake in hospitalized or community-dwelling older adults [4].

Regarding interventions to support adequate fluid intake in older persons, the literature search identified six additional studies, including two non-systematic reviews:

The aim of the invited review from Cook and co-authors [18] was to provide an overview of the literature on hydration interventions in care home residents, highlighting relevant areas for future research [18]. The authors reported some of the systematic reviews also referred to in the ESPEN guideline [4], which is probably the reason for the same conclusion, i.e., beverages intake in care home residents is too low, and no hydration intervention alone was effective in addressing the complex problems in older residents [18].

Another review focused on texture-modified food (TMF) and beverages in dementia and residential aged care facilities [6]. The authors concluded that there is a lack of evidence that TMF improves, among others, hydration and that adverse effects include poorer fluid intake [6]. Hence, texture-modified beverages, e.g., thickened liquids, do not seem to solve the problems with low-intake dehydration.

Jimoh and co-authors [19] performed an observational study among residents in nursing homes. In 22 residents, the fluid intake was directly observed by weighing and recording the intake of all drinks over 24 h. Most drinks were consumed between meals (59%, including 10% with medications). More than half of the participants (*n* = 12) achieved the European Food Safety Authority (EFSA) targets for drink intake, which are in line with the recommendations from ESPEN (see Table 1). Residents with sufficient compared to insufficient fluid intake drank more with medications and before breakfast. They were offered drinks more often, but drink variety did not differ [19]. This suggests that a more frequent offering of beverages may improve intake of fluid.

Marra and co-authors [20] performed another observational study among long-term-care residents. Hydration status was assessed by serum osmolality. Total fluid intake was quantified by weighing ingested food, beverage, water, and oral nutrition supplement (ONS). Total fluid intake positively correlated with caloric intake and drink consistency, with higher daily fluid intake in individuals consuming thin versus thickened liquids (90% of the subjects) [20]. Interestingly, no difference in total fluid intake was identified in relation to the number of daily between-meal snacks. In contrast to what could be expected, subjects with prescribed ONS had lower daily fluid intake compared to those without ONS prescription (1082 ± 411 mL/day vs. 1323 ± 446 mL/day, *p* = 0.001) [20]. These findings suggest that thin liquids should be recommended for both individuals with and without ONS to support older people to drink well.

Bak and co-authors [21] conducted a pre-post improvement project to identify characteristics of drinking vessels that best reflect nursing home residents needs’ and to evaluate the effect of drinking vessels with these characteristics on the fluid intake [21]. In the first part of the study, residents were served something to drink from standard and alternative vessels and asked to rate each vessel on ease of handling, volume, ease and pleasantness of drinking, and appearance. In the second part of the study, the vessels with the highest ratings were tested in a unit with 25 beds. The best-rated vessels were light, had large handles, and held 200–300 mL of fluid. The introduction of the new drinking vessels resulted in higher mean fluid intake at breakfast (139 ± 84 mL vs. 205 ± 12 mL; *n* = 65, *p* = 0.003) [21].

The same research group did another pre-post quality improvement study regarding hydration in care home residents through increasing opportunity to drink and choice of drinks [22]. The improvement activities included the following [22]:

1. Extending drinking opportunities comprised three interventions:
  - Pre-breakfast drinks: providing drinks to residents moved to the dining room prior to breakfast at one nursing home.
  - Drinks after meals: residents were offered hot drinks after lunch and dinner at another nursing home.
  - Protected Drinks Time (PDT): all residents were served a drink and where needed, provided with assistance to drink during the mid-afternoon drinks round at both nursing homes.
2. The choice of beverages was increased by developing a Drinks Menu. The menu provided support for residents when choosing a drink and encouraged staff to offer more than one drink. The Drinks Menu was combined with PDT and introduced in both homes.

The study was able to show that residents consume more beverages (before, with, and after meals) when they have the opportunity, choice, and support. Previous to the study, staff had expressed concerns that additional drinks would negatively affect the amount residents consumed at the next drinking opportunity, which proofed to be unfounded. The provision of additional structured drinking opportunities increased the number of residents receiving drinks and resulted in more fluids being consumed. The additional drinking opportunities primarily targeted independent residents, as they tended to be offered only to those in the dining room, and mainly residents who needed assistance benefited whilst PDT. Further research is necessary to extend this intervention to residents in their rooms, including ensuring adequate support with drinking [22]. The authors identified that several key factors influenced the success of changes in practice and subsequent sustainability of the interventions. These included allocation of staff to activities, availability of stock/equipment, establishing clear communication systems, and leadership of the care team [22]. All of these support the recommendations in the ESPEN guideline (see Table 1).

### 3.4. How Much Should Older People Drink Each Day?

As can be seen in Table 1, the ESPEN guideline recommends that older women should be offered at least 1.6 L and older men at least 2.0 L of drinks each day, unless a clinical condition requires a different approach. The ESPEN recommendation highlights that a different and more individual approach might be needed, as, e.g., larger individuals may require more fluid. Fluid losses due to extreme temperatures (e.g., summer heat), during or after physical activity, due to fever, diarrhea, vomiting or severe bleeding also need to be compensated by higher intake. On the other hand, e.g., older adults with heart and renal failure may need a restriction of fluid intake [4].

Regarding how much older people should drink, the literature search identified no more recent guidelines that addressed low-intake dehydration, but one critical review: Masot and co-authors [23] performed a review about fluid intake recommendations (in published articles and guidelines) for older adults. A literature search was conducted using PUBMED, Scopus, Cochrane, and Google Scholar until April 2020, focusing on people aged 65 years or older and on different care levels [23]. The authors suggest that most recommendations of international organizations do not consider the physiology of ageing or typical health problems of older adults. However, Masot and co-authors [23] concluded that older people should drink between 1.5 and 2.0 L/day. More specifically, they recommended to follow the ESPEN and EFSA guidelines, with ESPEN (Table 1) following EFSA recommendations.

### 3.5. What Should Older People Drink Each Day?

As can be seen in Table 1, the ESPEN guideline recommends that older people should consume appropriate (i.e., hydrating) drinks according to their preferences. For example, alcoholic beverages (e.g., beer and lager) can be hydrating and may be suitable for some older adults. However, alcohol consumption may need to be restricted for medical or social reasons. Despite concerns about the “dehydrating” effect, there is good evidence that coffee and alcoholic beverages (in adequate amounts) do not cause dehydration [4].

Regarding the type of drinks older people should drink each day, the literature search identified only one additional study. Polhuis and co-authors [24] conducted a randomized diet-controlled crossover trial to evaluate the diuretic action of weak and strong alcoholic beverages in older men. During the intervention, men received either beer; non-alcoholic beer; red wine; non-alcoholic red wine; spirits; or (tap) water. An equivalent amount of 30 g alcohol was administered [24]. The results showed that only moderate amounts of stronger alcoholic beverages (spirits) led to a temporary diuretic effect compared to their non-alcoholic counterparts. It is suggested, that the consumption of moderate amounts of a weak alcoholic beverage such as beer is safe in terms of hydration for elderly men. However, also the diuretic effect of stronger alcoholic beverages was small and short-lived, and therefore, it is suggested that the diuretic effect of moderate alcohol consumption—independent of the alcohol concentration—may be temporary and thus negligible in euhydrated older men [24].

### 3.6. Level of Evidence

The results of the rating of the level of evidence of the identified papers is presented in Table 2. For the majority of the cohort studies and the diagnostic accuracy studies the level of evidence was high. None of the three identified reviews were systematic and therefore rated as expert opinions (level 4) Unfortunately, it was not possible to assess the pre-post studies by means of the Scottish Intercollegiate Networks System (SIGN), as there is no checklist for such studies.

## 4. Discussion

The ESPEN guideline on nutrition and hydration in geriatrics, published in 2019, provides evidence-based recommendations and consensus on key issues regarding low-intake dehydration. In this narrative review, we identified 16 new publications mainly in line and supporting the recommendations (see Table 1). Only one article was contrary to the ESPEN guideline. It can be concluded, that low-intake dehydration in older people is a complex problem to address and the prevalence is still high, although it seems to be a rather simple problem of not drinking enough. Low-intake dehydration is rarely identified and it remains unclear how to best intervene to prevent or treat it.

In the following, the results identified in the current review are discussed in the context of the ESPEN recommendations.

### 4.1. How Should Low-Intake Dehydration Be Identified in Older Persons?

Considering the evidence from the ESPEN guideline as well as additionally identified literature within this narrative review, the use of calculated osmolarity should be enhanced. Bunn and Hooper suggested using osmolarity within a 2-stage screening process that includes serum osmolarity, calculated from sodium, potassium, urea, and glucose levels, followed by serum osmolality measurement for those identified as high risk (calculated serum osmolarity >295 mmol/L) [14].

Despite the described consensus of literature, there seems to be a need for further research regarding this measurement. A multidisciplinary group of experts has suggested, among others, the following research topics [1]:

- A prospective, interventional study that targets parameters of normal hydration (e.g., plasma osmolarities 280–300 mOsm/kg) and determines whether this translates to health and health economics co-benefits.
- The causality of the association between plasma osmolarity thresholds and adverse outcomes needs to be tested through interventional studies.
- The development of a suitable device for the routine, bedside assessment of plasma osmolality.

Furthermore, it should be considered that the measurement of serum osmolality is challenging outside the hospital and may not be possible in every country [4]. For example, in the nursing home, some laboratory tests cannot be taken and/or analyzed and the involvement of other laboratories would be required. In addition, there may be difficulties in drawing blood samples or conducting other assessments, i.e., due to cognitive impairments and time and staff constraints. This delays the commencement of treatment resulting in deterioration of residents’ health and avoidable hospital admissions [10].

To deal with this problem, a Delphi study was performed quite recently to reach a consensus on a relevant and feasible method (or combination of methods) to diagnose dehydration in nursing home residents [10]. The resulting strategy comprehends a presumption phase, where anamnestic items and physical symptoms are examined, followed by a confirmation phase with blood tests to confirm the diagnosis of low-intake dehydration [10].

A wide range of literature focused on identifying older adults that are already dehydrated, but less information is available regarding the identification of older adults with risk of dehydration and the prevention of low fluid intake [27]. To be able to intervene at an early stage, it may be useful to increase awareness of risk factors for low-intake dehydration. For example, the literature shows an overlap between the causes of malnutrition and low-intake dehydration in older people [28,29,30,31,32]. In relation to this, one must be aware that an apparently simple solution to the two problems, i.e., the offering of ONS, which provides both nutrients and fluids, might not always be the correct one. For example, Marra and co-authors found that subjects with prescribed ONS had lower total fluid intake than those without [20]. It is therefore important to recommend the intake of thin liquids when ONS is prescribed.

Regarding the identification of older adults with low-intake dehydration, it might be easiest to consider all older persons to be at risk and to encourage all to increase fluid intake. As the literature suggests, it is difficult to identify low-intake dehydration, especially among older adults in nursing homes, where it is seldom possible to measure e.g., serum osmolality [10]. This approach is also recommended in the ESPEN guideline as a Good Practice Point [4].

### 4.2. What Interventions May Help to Support Older Persons to Drink Well and Prevent Low-Intake Dehydration?

The updated literature search showed that there is still limited evidence supported by randomized controlled trials on which interventions may help older people to drink well. The available evidence is based on studies in nursing homes and hospitals where research is particularly difficult (time and staff constraints, cognitive and functional impairments of participants) [33].

Neither the ESPEN guideline [4] nor the updated literature search identified any published studies of interventions performed among community-dwelling older adults. As prevalence is assumed to be lower, low-intake dehydration might be even harder to identify or treat in this setting, not the least due to the apparent significant knowledge gaps among community-dwelling older people at risk of low intake dehydration (and malnutrition) identified by Bhanu and co-authors [34]. In addition to the prevalence of low-intake dehydration, also risk factors such as polypharmacy [7], functional and cognitive impairment [3,8], and voluntarily reduced fluid intake, appear to differ between the settings (hospital, nursing home, community-dwelling). To support older people to drink well, it might be essential to consider these different risk factors leading to low fluid intake, as customized and adjusted interventions might be favorable [27].

Further research on interventions to increase fluid intake could focus on several aspects, including beverage offer (frequency, timing, variety, and consistency), drinking vessels, staff awareness, technological possibilities, and the social aspects of drinking:

Based on the studies identified during the literature search, it appears that a more frequent and systematic offering of beverages may improve fluid intake [19,22]. Furthermore, there might be a need to focus on beverage consistency, as total fluid intake is higher in those consuming thin vs. thickened liquids [20]. Recently, it has been questioned whether thickened liquids have a place in the dysphagia tool kit at all [35]. Additionally, supporting and extending residents’ fluid choices (e.g., with the Drinks Menu) might positively influence the acceptance of offered drinks [22]. All these findings support the ESPEN recommendations.

One study identified in the literature search focused on drinking vessels, an aspect not mentioned in the ESPEN guideline. An obvious cause of limited intake of beverages is that the drinking vessels may be difficult to handle, e.g., too heavy for frail older people. Replacing these vessels with ones that are easier to handle appears to increase intake of beverages [21].

To improve fluid intake, it might be an opportunity to include nursing staff and care providers. Increasing staff awareness and especially knowledge regarding the detection of low-intake dehydration, interventions, as well as the adequate amount of fluid intake per day, seems essential [36,37]. Maybe not only by educating, but by actively increasing awareness and facilitation [38]. In relation to this, Paulis and co-authors [2] highlight the importance of differentiating between acute and chronic dehydration. The latter develops slowly after a longer period of inadequate fluid intake, whereas acute dehydration is a consequence of an illness. Subsequently, acute dehydration is more visible and predictable and could therefore be detected more easily [2]. However, this requires that nurses are aware of these different types of dehydration.

Further research is needed regarding adequate intervention strategies, for example with technological possibilities to monitor the intake of beverages: In the Cochrane review by Hooper and co-workers [8], drinks intake showed some potential on being useful to discover low-intake dehydration.

Unfortunately, the assessment of fluid intake in older adults is often highly inaccurate because drinks are omitted from staff assessments and because recordings refer to the amounts of beverages provided rather than the consumed amount [4].

Different innovative strategies have been tested, including, for example, bed weights, electronic charts and more, so far without definite results [39]. Hence, a reliable and feasible solution, especially for those frail older people where staff is not available around the clock, is needed. In the UK, a recently developed hydration monitoring app appears promising, but further development is needed mainly due to technical issues [40].

Furthermore, drinking (and eating) has a social aspect and as many older adults have reduced social contacts, it might be an opportunity to develop supporting interventions. According to caregivers’ perception, interventions focusing on the social aspect of food and fluid intake might be useful [36,37].

It might also be possible to focus on a combined intervention with technological possibilities and social involvement. Social involvement might be increased by virtual reality settings, technological gadgets and (serious) games [41,42]. Especially for older adults with cognitive impairment who might forget to drink [43], regular feedback regarding the amount of fluid intake might help to improve intake.

### 4.3. How Much Should Older People Drink Each Day?

The current narrative review did not identify any new findings regarding the amount of fluid that should be consumed. Therefore, until there is further research, the ESPEN/EFSA recommendations regarding the amount of fluid needed to prevent dehydration should be used. Communicating this amount to older adults, nursing staff. and other care providers (homecare, family, friends) is important to prevent low-intake dehydration.

### 4.4. What Should Older People Drink Each Day?

Considering the evidence from the ESPEN guideline as well as additionally identified literature within this narrative review, it seems to be more important that older people drink, not what they drink. However, consuming other beverages besides water, such as smoothies, and milk products, can increase the intake of nutrients in addition to hydration.

Regarding the intake of alcohol as part of fluid intake, it is necessary to distinguish between frequent, heavy alcohol consumption and light to moderate alcohol consumption, which appears to be safe in terms of hydration, at least in older men [24].

### 4.5. Limitations

Although a systematic approach was used to identify the literature and the search was focused, the included publications may not represent all available studies and reviews on the effects of low-intake dehydration and specific domains. It should be noted, that only one database was used, non-English publications and articles with missing full text were excluded, and the selection of articles was not performed in duplicate. In addition, although the search was based on the ESPEN guideline, there is a possibility that the search terms did not reflect all relevant aspects.

## 5. Conclusions

Hydration plays an important role in older adults. Despite its simple cause—inadequate intake of beverages—low-intake dehydration appears to be a very complex problem to address. Considering the evidence from the ESPEN guideline on which consensus was reached, as well as additionally identified literature within this narrative review, it is necessary to take setting specific differences and individual problems and needs into account (e.g., needing to be reminded of drinking, social environment, type of drink and vessel) to tackle low-intake dehydration in older adults. In general, the recently identified literature supports the recommendations communicated in the guideline. It is necessary to increase awareness of the prevalence and severity of low-intake dehydration among older adults and in nursing staff in care homes and hospitals as well as in carers of older adults living at home.

There is still a need for more research: firstly, regarding the identification of dehydrated older adults or those at risk within the clinical setting and with a practical focus, especially among community-dwelling older adults; secondly, regarding the adequate interventions to improve fluid intake; and thirdly, regarding the adequate amount of fluid intake.

## Figures and Tables

**Table 1:** Evidence-based recommendations regarding low-intake dehydration (adapted from [4]) §).

	Research Question (RQ)	Recommendation §	Grade of Recommendation *
**1**	**How should low-intake dehydration be identified in older persons?** **(RQ 1.1, 1.2, 1.3)**	An action threshold of directly measured serum osmolality > 300 mOsm/kg should be used to identify low-intake dehydration in older adults	B
		Where directly measured osmolality is not available then the osmolarity equation (osmolarity = 1.86 × (Na+ + K+) + 1.15 × glucose + urea + 14 (all measured in mmol/L) with an action threshold of >295 mmol/L) should be used to screen for low-intake dehydration in older persons	B
		Simple signs and tests commonly used to assess low-intake dehydration such as skin turgor, mouth dryness, weight change, urine color or specific gravity, shall NOT be used to assess hydration status in older adults.	A
		Bioelectrical impedance shall NOT be used to assess hydration status in older adults as it has not been shown to be usefully diagnostic	A
**2**	**What interventions may help to support older persons to drink well and prevent low-intake dehydration?**	To prevent dehydration in older persons living in residential care, institutions should implement multicomponent strategies across their institutions for all residents	B
		These strategies should include high availability of drinks, varied choice of drinks, frequent offering of drinks, staff awareness of the need for adequate fluid intake, staff support for drinking and staff support in taking older adults to the toilet quickly and when they need it.	B
		Strategies to support adequate fluid intake should be developed including older persons themselves, staff, management, and policymakers	A
**3**	**How much should older people drink each day?**	Older women should be offered at least 1.6 L of drinks each day, while older men should be offered at least 2.0 L of drinks each day unless there is a clinical condition that requires different approach	B
**4**	**What should older people drink each day?**	A range of appropriate (i.e., hydrating) drinks should be offered to older people according to their preferences	B

* See details for grades of recommendation in [4]. § ESPEN guideline contain two more evidence-based recommendations regarding treatment of low-intake dehydration (recommendation 72 and 73). The recommendation is to offer subcutaneous or intravenous fluids. As this is often not feasible outside the hospital, these two recommendations are not included.

**Table 2:** Characteristics of included studies.

RQ ^1^	Publication	Study Type *if Applicable: Population*	Relevant Findings	Level of Evidence ^2^	Consistency with ESPEN Guideline
**1.1**	**Lacey et al. 2019** **[1]**	**Expert opinion** *n = 12, experts of varying specialties*	- Plasma osmolality (in mOsm/kg) is an objective surrogate marker of low-intake dehydration - Osmolality is underutilized in clinical practice	4	Yes
**1.2**	**Lacey et al. 2019** **[1]**	See above	- When plasma osmolality cannot be assessed, the authors recommend to calculate plasma osmolarity (CO) (in mmol/L)	4	Yes
	**Munk et al. 2021** **[11]**	**Diagnostic accuracy study** *n = 90, older adults from emergency medical department*	- 32% with impending (≥295–300 mOsm/kg) and 11% with current dehydration (>300 mOsm/kg) - Significant association between CO and Osmolality - Using CO is superior to current clinical practice	2+	Yes
	**Woijszel et al. 2020** **[12]**	**Cohort study** *n = 358, hospitalized older adults*	- 58% with dehydration (CO > 295 mmol/L) - Dehydration was more frequent in patients with e.g., multimorbidity, polypharmacy, hypertension, diabetes, chronic kidney disease,	2+	Yes
	**Mantantzis et al. 2020** **[13]**	**Cohort study** *n = 1047, community-dwelling older adults*	- 33% with dehydration (CO > 296 mmol/L) - Higher CO was associated with older age, more morbidities, and greater decline in cognitive functioning and well-being over time	2+	Yes
**1.3**	**Bunn and Hooper 2019** **[14]**	**Diagnostic accuracy study** *n = 188, care home residents*	- Commonly used clinical signs and symptoms of low-intake dehydration (49 tested) inadequately discriminated between persons with or without low-intake dehydration - The authors suggest to use a 2-stage screening process instead of clinical signs and symptoms - 1: CO, 2: Serum osmolality measurement if CO > 295 mmol/L	2+	Yes
	**Lacey et al. 2019** **[1]**	See above	- Clinical signs and symptoms are not reliable outside extremes - They should not be used alone to detect dehydration	4	Yes
	**Johnson & Hahn 2018** **[15]**	**Cohort study** *n = 60, nursing home residents*	- 51% showed renal fluid conservation consistent with dehydration - Clinical signs of mucous membranes and tongue furrows did not correlate with serum osmolality - Clinical signs might rather reflect physical status and age than dehydration	2−	Yes
	**Akdeniz et al. 2018** **[16]**	**Cohort study** *n = 40, hospitalized older adults*	- Average fluid intake was 1747 mL/day - 20% were dehydrated and 23% had impeding dehydration, despite comparably high fluid intake - The clinical relevance of low fluid intake as a marker of dehydration is questionable	2+	Yes
	**Ekman et al. 2020** **[17]**	**Cohort study** *n = 38, rehabilitating older adults after hip surgery*	- Average fluid intake was 1008 mL/day - 18% with dehydration (≥300 mOsm/kg), those showed correlations to presence of tongue furrows and poor skin turgor - 21% with concentrated urine, those showed correlations to low fluid intake and a decreased body weight	2−	No
**2**	**Cook et al. 2019** **[18]**	**Literature review** *care home residents*	- Care home residents have low fluid intake - No hydration intervention alone was effective in addressing dehydration	4	Yes
	**Painter et al. 2017** **[6]**	**Literature review** *dementia and aged care facilities*	- Intake of texture-modified fluids was associated with lower energy and fluid intake - Lack of evidence that texture modified fluids improve fluid intake and negative consequences (i.e., aspiration, pneumonia)	4	Yes
	**Jimoh et al. 2019** **[19]**	**Cohort study** *n = 22, long-term care residents*	- Drinks are mostly consumed between meals - Residents with sufficient fluid intake were offered beverages more frequently and drank more with medications and before breakfast - Offering drinks more frequently might improve fluid intake	2-	Yes
	**Marra et al. 2016** **[20]**	**Cohort study** *n = 247, long-term-care residents*	- 31% with impending (295–300 mOsm/kg) and 38% with dehydration (>300 mOsm/kg) - Average water intake was 1147 mL/day - Variance in water intake was influenced by, e.g., type of liquid beverage (thin vs. thick), type of ONS, which could be targeted by nutritional interventions	2+	yes
	**Bak et al. 2018** **[21]**	**Pre-post study** *Two wards, nursing home residents*	- Drinking vessels which were lightweight, with large handles and volume of 200–300 mL were preferred - Introduction of new vessels at breakfast improved fluid intake	n.a.	Yes
	**Wilson et al. 2019** **[22]**	**Pre-post study** *Two care homes, Care home residents*	- Increased choice of beverages and opportunities to drink was associated with increased range of consumed fluid and higher fluid intake	n.a.	Yes
**3**	**Masot et al.** **[23]**	**Literature review** *Older adults at different care levels*	- Recommendations do not consider physiology of ageing and health problems of older people - Authors recommend 1.5–2.0 L of fluid per day	4	Yes
**4**	**Polhuis et al.** **[24]**	**Randomized Trial** *n = 20, elderly community-dwelling men*	- The intake of equal volumes of wine and spirits compared to non-alcoholic wine and water resulted in higher urine output within 4 h, but not 24 h - Moderate amounts of stronger alcoholic beverages showed short diuretic effects	1++	Yes

^1^ RQ1: How should low-intake dehydration be identified in older persons? 1.1 by osmolality, 1.2 by osmolarity, 1.3 by clinical signs and bioelectrical impedance (BIA). RQ2: What interventions may help to support older persons to drink well and prevent low-intake dehydration? RQ3: How much should older people drink each day? RQ4: What should older people drink each day? ^2^ Level of evidence according to the Scottish Intercollegiate Guidelines Network (SIGN) 1++ (highest), 4 (lowest) [4]. SIGN do not have tools for pre-post studies, so for two papers SIGN were not applicable (n.a.). CO = calculated osmolarity, BMI = body mass index, n.a. = not applicable, ONS = oral nutritional supplements, RQ = research question.
